# Location: root architecture structures rhizosphere microbial associations

**DOI:** 10.1093/jxb/erad421

**Published:** 2023-10-26

**Authors:** Tania Galindo-Castañeda, Martin Hartmann, Jonathan P Lynch

**Affiliations:** Department of Environmental Systems Service, ETH Zürich, 8092 Zurich, Switzerland; Department of Environmental Systems Service, ETH Zürich, 8092 Zurich, Switzerland; Department of Plant Science, The Pennsylvania State University, University Park, PA 16802, USA; University of Warwick, UK

**Keywords:** Carbon rhizodeposition, lateral roots, number of axial roots, rhizosphere microbiome, root growth angle, rooting depth, root system architecture, soil redox potential, soil vertical gradients

## Abstract

Root architectural phenotypes are promising targets for crop breeding, but root architectural effects on microbial associations in agricultural fields are not well understood. Architecture determines the location of microbial associations within root systems, which, when integrated with soil vertical gradients, determines the functions and the metabolic capability of rhizosphere microbial communities. We argue that variation in root architecture in crops has important implications for root exudation, microbial recruitment and function, and the decomposition and fate of root tissues and exudates. Recent research has shown that the root microbiome changes along root axes and among root classes, that root tips have a unique microbiome, and that root exudates change within the root system depending on soil physicochemical conditions. Although fresh exudates are produced in larger amounts in root tips, the rhizosphere of mature root segments also plays a role in influencing soil vertical gradients. We argue that more research is needed to understand specific root phenotypes that structure microbial associations and discuss candidate root phenotypes that may determine the location of microbial hotspots within root systems with relevance to agricultural systems.

## Teaser

Root architecture and soil vertical gradients create a diverse set of physicochemical conditions in which rhizosphere microbes live; however, these scaffolds of soil microbial ecology are rarely studied.

## Introduction

Suboptimal water and nutrient availability are primary, pervasive constraints to plant production in terrestrial ecosystems (Lynch, 2022a). Water, nitrogen, and phosphorus availability are commonly limiting in natural ecosystems and in low-input agroecosystems. In high-input agroecosystems, intensive fertilization and irrigation cause environmental degradation, and, in non-irrigated systems, drought stress is common and increasingly problematic as a result of global climate change. Root system architecture (RSA), the physical arrangement of root organs in space and time, determines the efficiency of soil resource capture, and therefore represents a potential solution to these current agricultural challenges.

RSA phenotypes such as root growth angle, rooting depth, lateral root branching density and length, and axial root number are possible crop breeding targets because they have adaptive value for soil resource capture (Lynch *et al.*, 2021), they are relatively easy to measure at high throughput (Trachsel *et al*., 2011; Colombi *et al*., 2015; Liu *et al*., 2021; Schneider *et al.*, 2021; Lynch, 2022b), and, for some, genetic control has been described (Ruangsiri *et al.*, 2021; Schneider *et al.*, 2021; Waite and Dardick, 2021). Other RSA-related phenotypes with adaptive value are dimorphism, the capability of roots to produce contrasting levels of a given trait in the same individual, and plasticity, the capacity to alter the phenotype in response to environmental stimuli (e.g. water deficit or suboptimal nutrient availability) (Lynch, 2019). These phenotypes are more complex to measure, and their genetics less well understood (Schneider and Lynch, 2020), but they integrate temporal and spatial dimensions into RSA.

While microbial effects on RSA have been widely documented, especially under controlled conditions (Eichmann *et al.*, 2021; Box 1), natural variation in root architecture of agricultural plants is rarely considered as an important factor determining root microbial associations. The influence of root phenotypes on microbial associations, and the synergies and trade-offs between plants and microbes when studying specific root architectural phenotypes with adaptive value for the capture of soil resources, have rarely been studied, especially under field conditions. Furthermore, the relative contribution of microbial feedbacks on the architectural phenotype of a plant under field conditions is unclear. Although plants under abiotic stress growing in artificial growth media recruit specific microbiomes that may help in acquiring more of the limiting resource (Castrillo *et al.*, 2017), aspects such as the localization and rate of microbial consumption of root exudates, and the metabolic cost of maintaining beneficial rhizosphere associations are not well understood either in controlled conditions or in the field.

Box 1. **Microbially driven changes in root architecture and relationship to nitrogen acquisition**

Several plant-growth promoting microorganisms (PGPM) cause changes in root architecture. Specifically, the production of lateral roots or the modification of the elongation rate of the primary root are usually reported in Arabidopsis (*Arabidopsis thaliana*) (Verbon and Liberman, 2016). The main mechanisms of these interactions are through the production of phytohormones by the microbial partner, or through the microbial modification in phytohormone perception by the plant (Frankenberger and Arshad, 2020; Verbon and Liberman, 2016). For example, several species of the soil fungus *Trichoderma* (Contreras-Cornejo *et al.*, 2009; Garnica-Vergara *et al.*, 2016) produce metabolites that interfere with auxin transporters in Arabidopsis and tobacco (*Nicotiana tabacum*). Also, *Pseudomonas putida* (Ortiz-Castro *et al.*, 2020), *Azospirillum brasilense* (Bashan and de-Bashan, 2010), *Bradyrhizobium japonicum* (Torres *et al.*, 2018), and *Burkholderia phytofirmans* (Zúñiga *et al.* 2013) produce indole-3-acetic acid that is, or becomes, active to induce lateral roots in their respective plant host. Furthermore, bacteria-grazing amoebas have been associated with increased levels of cytokinins under high concentrations of nitrate, and the cytokinins have been hypothesized to interact with increased free auxin product of the grazing of the amoeba in the rhizosphere of *Lepidium sativum* to increase lateral root branching density (Krome *et al.*, 2010). Nitric oxide (NO) has been discussed as a possible regulator of lateral root branching density (Singh *et al.*, 2019), which could then link NO to bacterial metabolism in the rhizosphere of crops. These are just a few examples of multiple PGPM reported in the literature to produce plant growth regulators that may participate in changes in root architecture and, therefore, indirectly in nitrogen uptake.

Although there is a notable amount of research on the potentialities and basic mechanisms of phytohormone-mediated plant–microbe interactions, the development of technologies based on such interactions in agroecosystems is still scarce. Usually, results observed in model plants such as Arabidopsis or on crops grown under controlled conditions are not reported equally under field conditions. Only a few reports demonstrate the utility of inoculation with PGPM to reduce nitrogen fertilization by causing changes in root architecture (see, for example, the example of *Azospirillum brasilense* and maize in Brazil by Hungria *et al.*, 2022). This reveals a poor understanding of such interactions, and the several questions that remain open (Galindo-Castañeda *et al.*, 2022; Verbon and Liberman, 2016). How and when do plant genetic determinants and plasticity in the production of new lateral roots interact with phytohormones produced (or modified) by microbes to control lateral root branching? If plasticity exists in lateral root branching patterns in response to nutrient patches in soil, is this only mediated by the plant directly, or is this a result of a feedback of root–microbe interactions in the rhizosphere? Possible cues to start changing root architectural patterns might originate from newly mineralized nitrogen, or just metabolized nitrogen-containing compounds by microbes. This is a fascinating, yet unexplored topic that merits further exploration at the field scale, using crops.

**Fig. 1.**
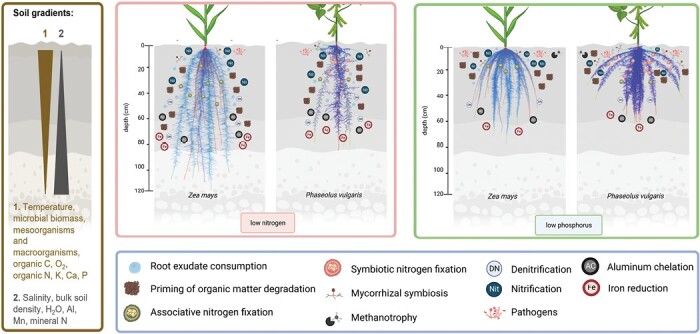
Hypothesized interactions of RSA with rhizosphere microbial processes (represented as icons) under limitation in a mobile (nitrate, Low N) and immobile (phosphorus, Low P) soil resource. Root models of the optimal root phenotypes to overcome each limitation for a cereal and a legume are depicted. The models are based on empirical observations (Lynch, 2019). Axial roots are in red and lateral roots in blue. The number and position of icons represent our hypothesized effects of the two RSA on the predominance and location of these rhizosphere processes along the soil profile and within the root system. Soil gradients are depicted to explain the hypothesized interactions. For example, shallow roots may favor nitrification, organic matter degradation, biodegradation of root exudates, and proliferation of fungal associations. In shallow roots, we hypothesize that nitrogen fixation would be performed mostly by bacteria with structures to protect nitrogenase from oxygen, and associative nitrogen fixation by a more diverse set of bacteria might be found in intermediate root systems. Deep roots would be associated with reductive microbial processes such as denitrification and ammonification, and manganese and iron reduction. Microbial aluminum chelation would be another process that might benefit the growing root tips in deep soil domains, although aluminum toxicity is also a problem in surface soil layers in tropical soils. More examples of similar analyses with RSA traits such as number of axial roots, dimorphism, and lateral root branching density can be found in ‘Interactions of root system architecture phenotypes with soil microbial processes’.

Here, we discuss RSA phenotypes that have adaptive value in crops (Lynch, 2022b) and how they might associate with microbes to favor specific microbial processes in the rhizosphere of crops interacting with vertical soil physicochemical gradients. We first present the context in which crop roots develop, the soil, and its vertical gradients. Next, we present facts and hypotheses regarding the interactions of RSA with carbon rhizodeposition, and then present primarily hypotheses on the rhizosphere microbial processes as determined by RSA (Fig. 1). We also present our perspective on pathogenic interactions with RSA. In this essay, we highlight recent advances in the understanding of microbial and root feedbacks as they relate to RSA (Box 2) and identify gaps resulting from a lack of integration of soil gradients and RSA in root–microbiome research. Further recommendations on how to move forward in this field are summarized in Box 3. Root growth rate and plasticity are important components of RSA that have been recently reviewed (Schneider and Lynch, 2020; Bonkowski *et al.*, 2021). However, they cannot be considered simple architectural traits, but rather complex processes that go beyond the scope of the present essay.

Box 2. **Key developments in locating rhizosphere microbial interactions considering RSA**

In the following studies, RSA is integrated with rhizosphere processes relevant to microbial processes. In (A), RSA had a significant effect on rhizosphere microbial communities. Microbial inoculation was significantly associated with changes in RSA through an auxin-dependent mechanism in maize (B) and in an auxin-independent mechanism in *A. thaliana* (C). Studies (D–F) show that RSA is linked to changes in exudates in ways that are relevant to microbial processes.

1. A. Kawasaki *et al.* (2021): the magnitude of differences in microbial diversity at root tips and root bases of wheat or rice are comparable with the variation in root microbial diversity between different plant species growing in the same soil.
2. B. Hungria *et al.* (2022): inoculation of maize with *Azospirillum brasilense* strains Ab-V5 and Ab-V6 allowed the reduction of 25% of fertilization overall in maize, in a set of 30 field trials spanning 10 years and contrasting levels of productivity, soil quality, soil organic carbon content, and tropical and subtropical conditions. The results are attributed to increases in root branching density, root hair length, root length, and specific root length, and without an increase in root biomass by means of the production of indole acetic acid (IAA). However, the evaluations of RSA were made in pots of <1 liter, where RSA is restricted.
3. C. Gonin *et al.* (2023): propose a new auxin-independent, ethylene-mediated mechanism by which microbes change lateral root branching density of Arabidopsis and *Selaginella moellendorffii*, which also seems to work under high salinity and iron deficiency.
4. D. McKay Fletcher *et al.* (2020): show that RSA is a better predictor than citrate exudation in phosphorus uptake in wheat.
5. E. Bilyera *et al.* (2021): provide an example of a specific interaction of a secondary metabolite (benzoxazinoid) reducing β-glucosidase activity in the rhizosphere, especially in mature root systems, where there is a higher activity of this enzyme.
6. F. Hosseini *et al.* (2022): exemplify the differences in rhizosphere activity of lateral versus axial roots under drought, finding that drought negatively affects the rhizosphere volumes of axial roots but not of lateral roots of wheat, while also favoring the activity of β-glucosidase but not of acid phosphatase, and leucine aminopeptidase.

Box 3. **Required tools, research questions, and prospective applications of integrating RSA with rhizosphere microbial processes**

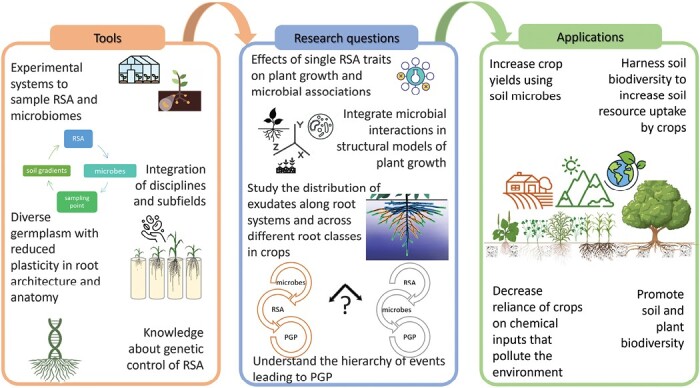

Research gaps in the study of relationships between RSA and rhizosphere microbes are presented in three categories: tools, research questions, and applications. We still lack tools to answer fundamental research questions to fulfill promising applications. PGP, plant growth promotion. Adapted from Galindo-Castañeda *et al.* (2022).

## Vertical gradients in soil

Soil is a heterogenous and vertically stratified matrix in which changing physical and chemical properties create distinct niches that influence root development and microbial activity. The arrangement of soil particles and aggregates into a porous structure that determines the availability of oxygen, water, and nutrients changes with depth (Hartmann and Six, 2023). Accumulation and decomposition of organic matter in the topsoil lead to the biogenic formation of stable micro- and macroaggregates that form a highly interconnected soil pore network, facilitating air permeability and water infiltration. As soil depth increases, biogenic processes of aggregate formation are replaced by physicogenic processes in which soil particles are packed into prismatic aggregates that form smaller and less interconnected pores, restricting the flow of oxygen, water, and nutrients (Koestel *et al.*, 2020; Pereira *et al.*, 2021).

Vertical physicochemical gradients influence microbial properties (He *et al.*, 2023). Topsoil domains characterized by greater diurnal and seasonal temperature variation, more oxygen, and less moisture favor aerobic microbes such as nitrifiers and methanotrophs as well as taxa able to tolerate fluctuating temperatures, desiccation, and solar radiation. Topsoil is also subjected to changes in pH due to fertilizer usage (Li *et al.*, 2020); therefore, acid-tolerant microbes would be better adapted in soils used for intensive agriculture. In contrast, deeper soil domains show increasing anaerobicity and soil moisture, favoring anaerobic microbial processes such as denitrification or iron reduction. The pH of deep soil domains varies from alkaline in limestone-derived soils, to acidic in highly weathered soils (Hartemink *et al.*, 2020). Plant nutrient availability also changes within the soil profile, with topsoils containing greater amounts of organic carbon and nitrogen as well as phosphorus, potassium, and calcium, while subsoils accumulate mineral nitrogen and have high bioavailability of manganese and aluminum (Lynch and Wojciechowski, 2015).

Agricultural practices such as tillage, fertilization, and irrigation often increase stratification. Although tillage homogenizes the topsoil, it also creates a plough pan that acts as a physical barrier for oxygen, water, nutrients, and possibly root penetration to deeper strata. Fertilizers, pesticides, and other amendments create local gradients that depend on solute mobility, for example with highly mobile mineral nitrogen accumulating in deeper strata and less mobile phosphorus residing in the topsoil (Lynch *et al.*, 2021). These vertical physicochemical gradients determine the presence and activity of microbial communities at a given location and drive their associations with roots. Moreover, vertical gradients also create differences in natural horizontal gradients that exist in soils and that directly create the rhizosphere environment, generating another layer to the interaction of root architecture with rhizosphere microbial activities. For example, the vertical gradient in oxygen content might create differences between the horizontal oxygen gradient of the rhizosphere of a root tip compared with the rhizosphere at the base of the same root, which might be in a shallower position across the soil profile. The former horizontal gradient is expected to be less pronounced than the latter.

## Carbon rhizodeposition and root system architecture

Carbon rhizodeposition includes root exudates, mucilage, shed root tissue, and debris from root loss (Dijkstra *et al.*, 2021). The location of carbon rhizodeposition within the root system under actual field conditions as well as the duration and functionality of the exuded or deposited compounds in field soils is poorly understood (Schnepf *et al.*, 2022; Zhang *et al.*, 2023). Specifically for root exudates and mucilage, the metabolic cost of their production is still a matter of research (Wen *et al.*, 2022). Understanding the spatiotemporal dynamics, rates of microbial consumption, and metabolic cost of carbon rhizodeposition is crucial to estimate the benefits of establishing plant–microbial associations to promote soil resource capture by plants (Galindo-Castañeda *et al.*, 2022).

Root exudates are produced in greater concentrations in root tips and the elongation zone, but differences in spatial patterns have been found in the production of enzymes and other compounds within root systems depending on mutations (Bilyera *et al.*, 2021), plant species (Razavi *et al.*, 2016), and stress conditions (Liu *et al.*, 2022; Smercina *et al.*, 2021). Besides root exudates, other sources of carbon such as enzymes (Hosseini *et al.*, 2022) and shedding of root tissue, especially from secondary root growth (Strock and Lynch, 2020), are found in the rhizosphere of mature roots. Traits such as root growth angle, rooting depth, and lateral root branching density determine the location and number of root tips, and therefore influence the abundance and location of root exudates and mucilage in reference to soil physicochemical gradients. Traits such as root diameter and the number of axial roots will modify rhizodeposition patterns around mature roots, as hypothesized in ‘Interactions of root system architecture phenotypes with soil microbial processes’. Anatomical phenotypes are also expected to interact with carbon rhizodeposition in mature root systems and with horizontal soil gradients (Galindo-Castañeda *et al.*, 2022). For example, root diameter, an anatomical trait affected by thickness and biochemical composition of the outermost layer of each root, is linked to root exudation and the composition of the rhizosphere microbial communities (Zai *et al*., 2021). Determining the associations between root architecture and carbon rhizodeposition would be especially important to predict resource utilization and the metabolic cost of soil exploration (Schäfer *et al.*, 2022).

The location of carbon rhizodeposition within root systems has important implications for resource capture and microbial activity in the rhizosphere in agricultural soils. The case of citrate exudate and phosphorus uptake by roots exemplifies this complexity. When the location and volume of rhizosphere hotspots and citrate-mediated phosphorus uptake are predicted using a model, contrasting results are found depending on whether such exudation occurs in the root tips only (in *Vicia faba*) or within the whole root system (in *Brassica napus*) (Schnepf *et al.*, 2022). These results link root architecture with root exudation *in silico* in a homogeneous soil matrix. If such models were integrated with vertical soil gradients of phosphorus availability, plants allocating exudates in the topsoil would probably have greater acquisition of phosphorus solubilized by citrate. Not surprisingly, RSA is highly correlated with citrate solubilization and is a better predictor of phosphorus uptake than citrate exudation itself (McKay Fletcher *et al.*, 2020).

If citrate or other exuded molecules are considered to estimate the extent of rhizosphere volumes, the extent and permanence of root exudates would vary depending on the root architecture and the rates at which microbes would consume the deposited carbon. This microbial degradation depends in large part on the depth at which these exudates are produced because the sorptive qualities of soils vary with depth, and because microenvironments change with depth (see ‘Vertical gradients in soil’). Therefore, root phenotypes such as lateral root branching density, root hairs, and root tips should be considered in models and estimations of rhizosphere volume and microbial activity.

The composition and permanence of root secreted compounds have effects on nutrient cycling and ecological interactions with microbes. On the one hand, plants encountering suboptimal availability of nitrogen and phosphorus change exudate composition (Smercina *et al.*, 2021; Wen *et al.*, 2022), and trigger changes in the nitrogen cycle in bulk soils (Liu *et al.*, 2022). How these processes change in the rhizosphere, where there is a microenvironment different from the bulk soil (Schnepf *et al.*, 2022), is unknown. On the other hand, the permanence of stress-responsive exudates in soils and the location in which they are produced are uncertain. A recent example of permanence of an exuded secondary metabolite is the production of benzoxazinoids, which mediate maize (*Zea mays*) defense against herbivores and seem to have a legacy and chemical fingerprint in field soils for a subsequent growth cycle (Gfeller *et al.*, 2023). No studies are available about the location of exudation of benzoxazinoids or many other compounds important for ecological interactions within the root system.

## Interactions of root system architecture phenotypes with soil microbial processes

Root architecture structures soil exploration in time and space and therefore regulates soil resource capture (Lynch, 2022b). Exploration of deep soil domains improves the capture of mobile resources such as water and nitrate in most environments, whereas topsoil foraging improves the capture of immobile resources such as phosphorus, potassium, and most other nutrients. Rooting depth is also related to biosequestration of atmospheric carbon, since carbon deposited in deep soil domains is degraded more slowly than that deposited in shallow soil (Kell, 2012). The extent and depth of root foraging for soil resources are also regulated by root anatomy, which determines the capacity of a root segment to acquire soil resources and transport them to the shoot (Lynch *et al.*, 2021). Root anatomy also regulates the metabolic cost of soil exploration, which is an important constraint to soil foraging (Lynch and Wojciechowski, 2015). In the following paragraphs we provide hypotheses on how RSA modifies microbial processes in the rhizosphere, which are also summarized in Fig. 1 and Table 1. Recent research supporting the development of this integrative field is presented in Box 2.

**Table 1.** Contrasting states of root architectural traits and the hypothesized effect on microbial habitat conditions

Root trait	Trait states	Hypothesized effect on root exudation, physicochemical conditions, and habitat space for microbes	Hypothesized effect on microbial processes in the rhizosphere	Supporting evidence
Rooting angle and depth	Steep—deep roots versus shallow—shallow roots	Increase in percentage of fixed carbon allocated to deeper soil layers. Increase of microaerophilic or anaerobic pockets around the rhizosphere of root tips. Soil compaction affecting the rhizosphere.	Increased number of root tips under microaerophilic conditions, favoring reduction of nitrogen compounds to ammonia or to N_2_. Nitrification may be favored in the rhizosphere of plants containing aerenchyma through which oxygen can pass into deeper soil domains. Nitrogen fixation may occur in the rhizosphere where air is brought to deep soil layers. The resulting ammonia might be quickly taken up by the plants rather than entering the dissimilatory N_2_ production. Associations with microbes tolerant to hypoxia, reduced rates of microbial consumption of root-derived carbon. Reduced predation by protozoa. Reduced exposure to soil-borne pathogens. Increased iron reduction and aluminum chelation.	**Not reported in the literature**—nitrogen cycle in paddy rice as it relates to soil depth: Ishii *et al*. (2011). Soil compaction effects on rhizosphere microbiota in crops: Longepierre *et al*. (2021, 2022)
Number of axial roots	Many versus few	Increased amounts of root exudates. Increased surface for microbial attachment. Larger amounts of substrate to be degraded from root decay. Steeper gradients of nutrient concentration in the depletion zone in the horizontal axis.	Overall increase in microbial abundance and diversity. Diversification in metabolism under more pronounced nutrient and water gradients in the depletion zones around the roots. Endophytic colonization increased. Reduced ammonia availability due to plant uptake might favor competence with microbes. Oxygen brought with the roots (either during or after root growth) might trigger nitrification of carbon sources such as root exudates or other sources such as debris or organic matter.	**Not reported in the literature**—review on plant–microbe competence for nitrogen: Moreau *et al.* (2015)
Lateral root density	High versus low	Greater number of exudation points from lateral roots. Greater number of attachment points for microbes. Steeper gradients of nutrient concentration in the depletion zone in the horizontal axis.	Overall increase in microbial abundance and diversity. Diversification in metabolism under more pronounced nutrient and water gradients in the depletion zones around lateral roots. Endophytic colonization increased. Reduced ammonia availability due to plant uptake might favor competition with microbes. Increased mycorrhizal colonization.	Schmidt *et al.* (2018); review on plant–microbe competence for nitrogen: Moreau *et al.* (2015) Studies on lateral root versus axial root: Saleem *et al.* (2016; Zai *et al.* (2021)
Lateral root length	Long versus short	Root exudates allocated further from the axial root, or from roots of lower branching orders. Overall increase of exudates per plant.	Differences in composition of the lateral root microbiome given the different horizontal gradients of resources between the two phenotypes. Longer roots may recruit a more diverse microbiome due to the increased soil volume explored. Attenuated effect of intra-root competition for nitrogen, which may lead to reduced plant–microbe competition for nitrogen	**Not reported in the literature**. Studies on lateral root versus axial root: Saleem *et al.* (2016); Zai *et al.* (2021); Kawasaki *et al.* (2021)

Modified from Galindo-Castañeda *et al.* (2022).

### Root growth angle and rooting depth

The growth angle of axial roots is perhaps one of the most important phenotypes determining the rhizosphere microbiome because axial roots are the scaffolds from which lateral roots emerge, therefore determining the depth at which most root biomass and exuding root tips are located. Such root allocation integrated with soil vertical gradients create diverse microbial niches (Fig. 1). Whether shallow roots or deep roots are associated with specific microbial processes in the rhizosphere remains largely unexplored. Mycorrhizal associations are more abundant in the basal segments of axial roots in common bean (*Phaseolus vulgaris*) under limiting phosphorus, but the symbiosis disappears with time due to secondary growth (Strock *et al.*, 2018). We expect that shallow roots favor nitrification, organic matter degradation, biodegradation of root exudates, and proliferation of fungal associations; and nitrogen fixation would be carried out mostly by bacteria with structures to protect nitrogenase from oxygen. Perhaps associative nitrogen fixation by a more diverse set of bacteria might be found in intermediate root systems, where oxygen is reduced (Fig. 1). Deep roots would be associated with reductive microbial processes such as denitrification and ammonification, and manganese and iron reduction. Microbial aluminum chelation would be another process that might benefit growing root tips in deep soil domains, although aluminum toxicity is also a problem in surface soil layers in tropical soils.

### Dimorphic root systems

An individual plant can have both shallow and deep roots, or have intermediate states between these two extremes. This can be advantageous when both immobile and mobile soil resources are limiting and the construction and maintenance cost must be minimized (Lynch, 2022b). In this case, diverging microbial communities might associate in the rhizosphere depending on the depth and root architecture. Carbon rhizodeposition produced in shallow soil domains would prime organic matter degradation and ammonia production, phosphorus solubilization, mycorrhization, and in some cases pathogenesis (see ‘Pathogenic associations and root system architecture’). Deep roots would meanwhile be surrounded by a less active microbial community participating in reductive reactions of metal and organic matter. Intermediate root systems with fanned root architectures might favor associative nitrogen fixation in the rhizosphere by protecting it from high oxygen concentrations that are present close to the surface.

### Number of axial roots

For microbial associations, roots with more axial roots represent increased colonization area during root elongation, and perhaps increased carbon deposition through exudates and root debris. More axial roots also means more lateral roots (Strock *et al.*, 2021), which are readily colonized by microbes. In crops such as maize and bean, expressing fewer axial roots is an adaptive phenotype under low nitrogen availability and water deficit, while being deleterious under low phosphorus (Saengwilai *et al.*, 2014; Gao and Lynch, 2016; Rangarajan *et al.*, 2018, 2022; Sun *et al.*, 2018; Schäfer *et al.*, 2022). Under soil impedance, an increased number of axial roots that can penetrate the hardpan in agricultural soils is beneficial, and overall enhances rooting depth and access to mobile resources (Strock *et al.*, 2021). The location of axial roots deeper in compacted soils might favor microbial species involved in the reduction of nitrate, sulfate, or iron to interact in the rhizosphere. For example, soil compaction caused an increase in denitrifiers in the rhizosphere of peas (*Pisum sativum*) and wheat (*Triticum aestivum*) (Longepierre *et al.*, 2022), and was associated with increased abundance of anaerobic prokaryotes and saprotrophic fungi, concomitant with decreases in aerobic prokaryotes and plant-associated fungi (Longepierre *et al.*, 2021).

There are other indirect effects of increased axial root number in crops. First, with more axial roots, intra-plant competition for resources might create steeper horizontal gradients of resources in the rhizosphere given the more numerous active root segments at a given depth, which may create a more selective niche for the microbiome, with microbial communities showing perhaps a more diverse spectrum in resource usage. Under such conditions, competition for resources can also occur between plants and microbes (Hill and Jones, 2019). Secondly, plants with more axial roots must use carbon more efficiently, given the high metabolic cost of axial roots (Rangarajan *et al.*, 2022). Therefore, carbon exudation in plants with many axial roots might be compromised, with direct consequences for the microbial commensals relying on such exudates. These processes have received little attention in the field of root microbiology.

### Lateral root branching density

Plants have the capability to branch profusely in response to nutrient patches in soils (Schneider and Lynch, 2020), and this response may be enhanced by microbes given their ability to trigger lateral root branching (Chiu *et al.*, 2022; Gonin *et al.*, 2023; Verbon and Liberman, 2016). This seems beneficial to the microbes because it creates more opportunities to associate with roots and it may increase the production of fresh exudates to feed these microbes. However, the utility of a microbially mediated increase in lateral root branching for the plant is debatable, because under some conditions the metabolic cost of lateral roots exceeds the benefits they afford for resource capture. For example, the best root phenotypes of maize and bean to acquire mobile resources that are more abundant in deep soil domains such as nitrate and water have reduced lateral root branching density (LRBD) (Fig. 1). The reason is that the construction and maintenance costs of more lateral roots exceed the increase in nitrogen or water uptake due to intra-root competition for these limiting resources, and that reduced production of lateral roots permits more internal resources to be available for axial root elongation into deeper soil domains (Lynch, 2022b). Therefore, we call for critical assessments of the advantages of forcing plants to produce more lateral roots through microbial inoculation, especially under stress conditions where mobile resources are highly limiting. On the contrary, plants growing under phosphorus limitations may benefit from associations with microbes, causing increased LRBD (Chiu *et al.*, 2022), because this phenotype increases phosphorus uptake through topsoil foraging (Jia *et al.*, 2018; Rangarajan *et al.*, 2022). Similar effects would be expected with other mobile and non-mobile resources within the soil profile. We call for more studies where inoculation experiments are accompanied by RSA assessments.

### Seedling roots

The seed microbiome influences the seedling microbiome (Matsumoto *et al.*, 2021; Walsh *et al.*, 2021), which probably interacts with root phenotypes in seedling establishment. Young root systems have different architectures compared with their mature counterparts (Perkins and Lynch, 2021), which corresponds to different availabilities of soil resources in agricultural systems at each phenological stage (e.g. nitrogen fertilizer, water, and tillage). Not surprisingly, the seedling microbiome is different from those of mature root systems (Tannenbaum *et al.*, 2020; Quiza *et al.*, 2023), with soil microbes having stronger effects than the resident seed microbiome in the assembly of the seedling root microbiome (Rochefort *et al.*, 2021). Conversely, most root microbiome research is frequently performed at the seedling stage and in small pots. Under these conditions, the seed microbiome might have a stronger legacy effect on the whole root microbiome as a result of the physical proximity of old and young roots. Therefore, the effects of microbial inoculation observed in small pots become irreproducible when scaling to field conditions. We call for studies with larger pots (mesocosms) with volumes comparable with those available to field-grown plants when field evaluation of seedlings is not possible.

## Pathogenic associations and root system architecture

Abiotic stress is a nearly universal constraint in terrestrial ecosystems, and plants generally experience multiple abiotic stress conditions concurrently (Lynch, 2022a). These stresses reduce plant health, growth, and fitness, and hence change their sensitivity to biotic stress. The range of potential interactions of abiotic and biotic stresses is complex. In some cases, abiotic stress decreases sensitivity to biotic stress, as is the case, for example, for nitrogen deficiency reducing the severity of obligate fungal pathogens and some foliar-feeding insects by reducing the nutritional quality of the host plant (Marschner, 1995). In other cases, resistance to biotic and abiotic stress shares a mechanistic basis, as is the case for silicon nutrition regulating plant response to pathogens, insects, and heavy metal toxicity (Etesami and Jeong, 2018). However, the general case is that abiotic stress weakens defense mechanisms and increases susceptibility to biotic stress (Suzuki *et al.*, 2014). Root architectural phenotypes capable of improving the capture of soil resources, thereby alleviating water and nutrient deficit stress, which are globally the most important abiotic stress factors (Lynch, 2022a), should therefore have an indirect benefit for resistance to biotic stress. A recent *in silico* study showed that root architecture directly affects plant growth in response to root loss (as occurs in response to many soil pests and pathogens) under nutrient stress, with some architectural phenotypes being resilient to root loss under nitrogen deficiency, or even increasing plant growth in response to root loss (Schäfer *et al.*, 2022).

Identifying RSA phenotypes that reduce the deleterious effects of root disease should be a target of plant breeding. Pathogen colonization can occur in both axial and lateral roots, causing root loss or diminishment of root functions (Galindo-Castañeda *et al.*, 2019). Crops are less sensitive to lateral root loss than to axial root loss (Schäfer *et al.*, 2022), and lateral roots are more sensitive to pathogen colonization. Therefore, plants with an RSA with a greater length ratio of axial to lateral roots might be more resilient to pathogen attack than plants with lower ratios.

Root growth angles that locate larger proportions of the root biomass in intermediate and deep soil domains rather than shallow domains may be beneficial to avoid pathogen colonization of fungi and pathogens that remain from previous seasons (Fig. 1). Deep or intermediate roots would be less exposed to the initial inoculant and, in general, hypoxic conditions reduce a wide range of soil-borne pathogens (Lopes *et al.*, 2022). There are very few field studies focusing on RSA and root pathogens, but phenotypes with deeper roots had increased disease resistance in alfalfa (Mattupalli *et al.*, 2019) and in durum wheat (Alahmad *et al.*, 2020; Saad *et al.*, 2023) for example.

To reduce deleterious effects of pathogen colonization, timing is important. Plants need time to develop resistance to certain pathogens. Therefore, adaptations to evade pathogen colonization at the beginning of the growth cycle might be advantageous. Seminal roots with steep root growth angles to reduce encounters with pathogens, rapid proliferation of lateral roots that might act as traps for pathogens to trigger resistance responses in the plant and facilitate the expression of immunity, and plasticity to rapidly replace lost roots with new lateral or axial roots could be examined as possible architectural phenotypes to reduce soil-borne disease in roots.

## Conclusion

RSA drives the location and environmental conditions in which root microbial associations occur in agricultural soils. However, RSA and soil vertical gradients are rarely included in root microbiome studies, creating a significant gap in our understanding of the mechanisms of root–microbe associations in real soils. Moreover, RSA has adaptive value for soil resource uptake, and plants naturally balance their carbon economy to allocate roots in soil domains where resources are more abundant. This allocation affects microbial processes in the rhizosphere in ways that are poorly understood. Therefore, efforts to select microbiomes that could benefit crops should include better ways to assess the mechanisms underlying root–microbiome associations using experimental systems that allow measurement of RSA and that account for soil vertical gradients. In this way, plants could express their real adaptations to acquire soil resources while also associating with soil microbes.

Research on the location of root carbon rhizodeposition within root systems is needed to understand microbial processes in the rhizosphere under realistic heterogenous soil conditions. We have provided several hypotheses regarding the effects of the interaction of RSA and soil vertical gradients on rhizosphere microbial associations and processes that merit experimental investigation (Table 1). Notably, research on the interactions of rhizosphere microbial processes with RSA is yet to be developed. We have further proposed a research agenda that will progressively lead to a more sustainable use of plant germplasm, soil microbes, and soil resources (Box 3). Methodological tools such as accessible experimental systems, sampling methods to capture the biodiversity of the rhizosphere in root systems, germplasm with contrasting but stable root phenotypes, and understanding of the genetic control of RSA, including plasticity, are urgent matters that will help achieve the goals of modern agriculture (Box 3).
